# Supplementary material for: Effect of water activity on rates of serpentinization of olivine
Source: Nat Commun. 2017 Jul 14;8:16107. doi: 10.1038/ncomms16107 (PMC5594423; doi:10.1038/ncomms16107)
Supplement: Supplementary Information [file ncomms16107-s1.pdf]

File name: Supplementary Information
Description: Supplementary Tables

File name: Supplementary Data 1
Description: All the data from the experimental runs. FPD, salinity and H2O consumed.

File name: Supplementary Data 2
Description: Step by step methodology to calculate the amount of H2O consumed from the FPD observations.

File name: Peer Review File
Description:

Supplementary Table 1 | Electron microprobe analysis of the olivine host.

	Concentration (wt.%)
SiO_2	40.67
MgO	50.03
CaO	0.05
MnO	0.126
FeO	8.66
NiO	0.37
Total	99.92
Mg #	85*

$$^*Mg\# = \left(\frac{MgO}{MgO+FeO}\right) \times 100$$

Supplementary Table 2 | TOF-SIMS data and composition of the reaction products of the SFI shown in Figures 2 and 3. The areas refer to the areas highlighted in the fluid inclusion shown in Figure 3.

Elements[a]	Region I		Region II		Region III		Region IV		Region V		Region VI		Region VII	
	Olivine	Brucite	Olivine	Serpentine	Olivine	Serpentine	Olivine	Serpentine	Olivine	Serpentine	Olivine	Brucite	Olivine	Serpentine
Na+	615	53677	1554	37148	1155	38146	1209	36086	985	82079	453	3165	2330	18358
^{25}Mg+	146296	103126	305704	80630	369753	14684	162388	112338	111957	116932	141797	9493	542468	74543
Si+	52707	65267	152903	56945	231141	8658	60456	57906	29471	34329	81683	6756	280256	54682
Fe+	47829	30132	107244	20184	144535	3831	52901	30230	43481	29408	52226	3039	192260	20500
Mg/Mg+Fe	0.75	0.77	0.74	0.80	0.72	0.79	0.75	0.79	0.72	0.80	0.73	0.76	0.74	0.78
Mg #	85[b]	87[c]	85[b]	92	85[b]	94	85[b]	90	85[b]	94	85[b]	88	85[b]	90

[a]TOF-SIMS data units are expressed in counts generated per ROI (region of interest).
[b]Mg # of the olivine was calculated from the EMPA data of Supplementary Table 1.
[c]Mg# of the ROI calculated from the cross-multiplication of the ^{25}Mg/(^{25}Mg+Fe) ratios and the Mg# of the host.
The mineralogy of the ROI was previously identified by Raman (Fig. 2).

Supplementary Table 3 | Rates of reaction, fluxes, activities, and residual data for individual SFIs from the experiments with starting compositions of H_2O-NaCl-$MgCl_2$ (Na/Mg 8:1).

Salinity (wt.%)	SFI	r (mol/sec)	J (mol/m^{2}sec)	log r	log J	a_{H_2O}	log a_{H_2O}	log J Residuals*	ξ
1	FIA 1A	9.56E-18	3.04E-08	-17.02	-7.52	0.995	-0.0021	-0.103	0.54
1	FIA 1B	8.10E-18	2.58E-08	-17.09	-7.59	0.995	-0.0021	-0.175	0.45
1	FIA 1C	7.49E-18	2.39E-08	-17.13	-7.62	0.995	-0.0021	-0.209	0.54
1	FIA 1D	8.90E-18	2.83E-08	-17.05	-7.55	0.995	-0.0021	-0.134	0.50
1	FIA 1E	8.90E-18	2.83E-08	-17.05	-7.55	0.995	-0.0021	-0.134	0.50
1	FIA 1F	9.56E-18	3.04E-08	-17.02	-7.52	0.995	-0.0021	-0.103	0.54
1	FIA 1G	6.03E-18	1.92E-08	-17.22	-7.72	0.995	-0.0021	-0.303	0.45
1	FIA 1H	8.90E-18	2.83E-08	-17.05	-7.55	0.995	-0.0021	-0.134	0.50
1	FIA 1I	8.90E-18	2.83E-08	-17.05	-7.55	0.995	-0.0021	-0.134	0.50
3.5	FIA 1A	4.23E-18	1.35E-08	-17.37	-7.87	0.984	-0.0069	0.120	0.48
3.5	FIA 1B	3.70E-18	1.18E-08	-17.43	-7.93	0.984	-0.0069	0.062	0.41
3.5	FIA 1D	1.76E-18	5.60E-09	-17.75	-8.25	0.984	-0.0069	-0.261	0.16
3.5	FIA 1H	2.23E-18	7.09E-09	-17.65	-8.15	0.984	-0.0069	-0.159	0.29
3.5	FIA 1I	2.46E-18	7.84E-09	-17.61	-8.11	0.984	-0.0069	-0.115	0.16
3.5	FIA 2C	4.00E-18	1.27E-08	-17.40	-7.89	0.984	-0.0069	0.096	0.16
3.5	FIA 2D	6.03E-18	1.92E-08	-17.22	-7.72	0.984	-0.0069	0.274	0.16
3.5	FIA 2E	5.45E-18	1.74E-08	-17.26	-7.76	0.984	-0.0069	0.230	0.34
3.5	FIA 3A	3.31E-18	1.05E-08	-17.48	-7.98	0.984	-0.0069	0.013	0.36
3.5	FIA 3B	3.81E-18	1.21E-08	-17.42	-7.92	0.984	-0.0069	0.075	0.16
3.5	FIA 3C	3.03E-18	9.66E-09	-17.52	-8.02	0.984	-0.0069	-0.024	0.16
3.5	FIA 3D	3.84E-18	1.22E-08	-17.42	-7.91	0.984	-0.0069	0.078	0.48
3.5	FIA 3E	4.14E-18	1.32E-08	-17.38	-7.88	0.984	-0.0069	0.110	0.34
3.5	FIA 3G	3.22E-18	1.03E-08	-17.49	-7.99	0.984	-0.0069	0.002	0.32
3.5	FIA 3H	3.14E-18	1.00E-08	-17.50	-8.00	0.984	-0.0069	-0.009	0.44
3.5	FIA 3I	3.44E-18	1.10E-08	-17.46	-7.96	0.984	-0.0069	0.030	0.54
3.5	FIA 3J	3.79E-18	1.21E-08	-17.42	-7.92	0.984	-0.0069	0.072	0.48
3.5	FIA 3K	3.72E-18	1.19E-08	-17.43	-7.93	0.984	-0.0069	0.065	0.52
3.5	FIA 3L	5.03E-18	1.60E-08	-17.30	-7.80	0.984	-0.0069	0.195	0.50
3.5	FIA 3M	5.04E-18	1.60E-08	-17.30	-7.79	0.984	-0.0069	0.196	0.29
3.5	FIA 3N	4.84E-18	1.54E-08	-17.31	-7.81	0.984	-0.0069	0.179	0.51
3.5	FIA 3O	2.27E-18	7.23E-09	-17.64	-8.14	0.984	-0.0069	-0.150	0.49
3.5	FIA 3P	2.66E-18	8.48E-09	-17.57	-8.07	0.984	-0.0069	-0.081	0.50
3.5	FIA 3Q	2.18E-18	6.94E-09	-17.66	-8.16	0.984	-0.0069	-0.168	0.51
3.5	FIA 3R	3.90E-18	1.24E-08	-17.41	-7.91	0.984	-0.0069	0.085	0.53
3.5	FIA 3S	4.45E-18	1.42E-08	-17.35	-7.85	0.984	-0.0069	0.142	0.49
3.5	FIA 3T	3.42E-18	1.09E-08	-17.47	-7.96	0.984	-0.0069	0.028	0.52
3.5	FIA 3U	3.71E-18	1.18E-08	-17.43	-7.93	0.984	-0.0069	0.063	0.49
3.5	FIA 3Y	4.97E-18	1.58E-08	-17.30	-7.80	0.984	-0.0069	0.190	0.28
3.5	FIA 3Z	3.03E-18	9.66E-09	-17.52	-8.01	0.984	-0.0069	-0.024	0.33
3.5	FIA 3AB	4.33E-18	1.38E-08	-17.36	-7.86	0.984	-0.0069	0.130	0.20
3.5	FIA 3AC	4.86E-18	1.55E-08	-17.31	-7.81	0.984	-0.0069	0.180	0.35
3.5	FIA 3AE	2.66E-18	8.48E-09	-17.57	-8.07	0.984	-0.0069	-0.081	0.48
3.5	FIA 4A	4.56E-18	1.45E-08	-17.34	-7.84	0.984	-0.0069	0.153	0.43
3.5	FIA 4D	3.84E-18	1.22E-08	-17.42	-7.91	0.984	-0.0069	0.079	0.43
3.5	FIA 4E	3.70E-18	1.18E-08	-17.43	-7.93	0.984	-0.0069	0.062	0.12
3.5	FIA 4F	2.56E-18	8.17E-09	-17.59	-8.09	0.984	-0.0069	-0.097	0.10
3.5	FIA 4G	1.92E-18	6.11E-09	-17.72	-8.21	0.984	-0.0069	-0.223	0.12
6	FIA 1A	1.04E-18	3.32E-09	-17.98	-8.48	0.974	-0.0116	0.077	0.26
6	FIA 1B	1.18E-18	3.75E-09	-17.93	-8.43	0.974	-0.0116	0.130	0.12
6	FIA 1E	9.81E-19	3.12E-09	-18.01	-8.51	0.974	-0.0116	0.051	0.07
6	FIA 1F	1.08E-18	3.45E-09	-17.97	-8.46	0.974	-0.0116	0.093	0.03
6	FIA 1G	9.36E-19	2.98E-09	-18.03	-8.53	0.974	-0.0116	0.030	0.25
6	FIA 1H	9.35E-19	2.98E-09	-18.03	-8.53	0.974	-0.0116	0.030	0.28
6	FIA 1I	1.10E-18	3.51E-09	-17.96	-8.46	0.974	-0.0116	0.101	0.33
6	FIA 2A	1.23E-18	3.92E-09	-17.91	-8.41	0.974	-0.0116	0.149	0.24
6	FIA2C	9.82E-19	3.13E-09	-18.01	-8.50	0.974	-0.0116	0.051	0.29
6	FIA 2H	1.11E-18	3.53E-09	-17.96	-8.45	0.974	-0.0116	0.103	0.16
10	FI A	6.80E-20	2.17E-10	-19.17	-9.66	0.958	-0.0187	-0.254	0.05
10	FI B	6.80E-20	2.17E-10	-19.17	-9.66	0.958	-0.0187	-0.254	0.05
10	FI C	1.33E-19	4.22E-10	-18.88	-9.37	0.958	-0.0187	0.035	0.09
10	FI D	8.38E-20	2.67E-10	-19.08	-9.57	0.958	-0.0187	-0.163	0.06
10	FI K	1.18E-19	3.74E-10	-18.93	-9.43	0.958	-0.0187	-0.017	0.08
10	FI L	6.80E-20	2.17E-10	-19.17	-9.66	0.958	-0.0187	-0.254	0.05

*These data represent the difference from the measured log J and the value predicted by the rate equation (Fig. 4A) in main text.

r is the rate of H_2O consumption in moles per second.

J is the flux of H_2O from the liquid phase into the product phases.

a is the activity of H_2O obtained using Eq3/6 software.

ξ = Extent of reaction calculated from the fraction of H_2O consumed.
